# First Experience With Catheter-Directed Thrombolysis for Massive Pulmonary Embolism at Teaching Hospital Jaffna, Sri Lanka: A Case Report

**DOI:** 10.7759/cureus.68270

**Published:** 2024-08-31

**Authors:** Kajananan Sivagurunathan, Pirannavan Ratnasingam, Dilan Jayanthan, Nalayini Jegathesan, Peranantharajah Thampipillai

**Affiliations:** 1 Internal Medicine, Teaching Hospital Jaffna, Jaffna, LKA; 2 Interventional Radiology, Teaching Hospital Jaffna, Jaffna, LKA

**Keywords:** trauma and pulmonary embolism, interventional radiology, reperfusion, pulmonary embolism, catheter-directed thrombolysis

## Abstract

Catheter-directed thrombolysis (CDT) is one of the modes of treatment for massive pulmonary embolism (PE). This case report shares the new experience of CDT for massive PE at Teaching Hospital Jaffna, Sri Lanka. A 54-year-old woman developed massive PE two days after a traumatic tibial fracture. She was hemodynamically unstable with hypotension and hypoxemia. The multidisciplinary team decided to go for CDT, administering alteplase. Follow-up imaging demonstrated complete thrombus resolution and significant clinical improvement. This case emphasizes the efficacy and safety of CDT for massive PE, particularly in patients at high risk for bleeding. Our experience at Teaching Hospital Jaffna accentuates the significance of individualized treatment strategies and the adoption of advanced techniques in resource-limited settings.

## Introduction

Pulmonary embolism (PE) is a common and fatal condition. In epidemiological studies, the annual incidence of PE varies between 39 and 115 cases per 100,000 people [1]. Massive PE is characterized by hypotension and right ventricular dysfunction, leading to cardiogenic shock with hypoxemia.

Managing massive PE presents significant challenges due to several factors, including hemodynamic instability, the high bleeding risk associated with treatment options, and the urgency of achieving an accurate diagnosis. Treatment selection is complex, requiring careful consideration of the risks and patient-specific factors such as bleeding tendencies. Additionally, resource limitations may constrain available interventions. Effective post-treatment monitoring and the necessity for multidisciplinary coordination further complicate the management of massive PE.

Conventional systemic thrombolysis is a mainstay of treatment for massive PE, aiming to dissolve the thrombus and restore pulmonary perfusion. However, systemic thrombolysis carries a high risk of major bleeding, particularly in patients with recent trauma or surgery. In such circumstances, catheter-based therapies, such as mechanical thrombectomy and catheter-directed thrombolysis (CDT), have emerged as promising alternatives. CDT delivers thrombolytic agents directly to the site of the thrombus, thereby reducing systemic exposure and the associated risk of bleeding [2,3].

Here, we present the case of a 54-year-old woman who had massive PE following a traumatic tibial fracture. Catheter-based therapy was chosen instead of systemic treatment due to the associated bleeding risk with a history of trauma. This case emphasizes individualized treatment approaches and the efficacy of CDT in managing massive PE.

## Case presentation

**Figure 1:**
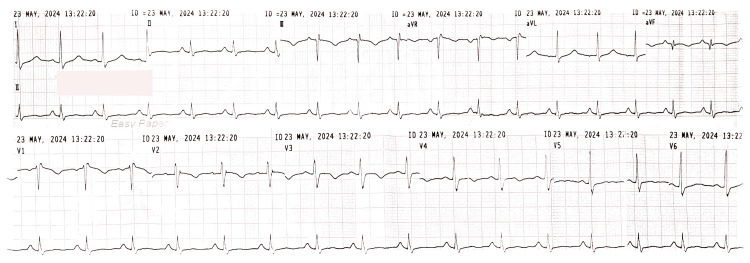
Electrocardiogram shows SI QIII TIII pattern—deep S wave in lead I, Q wave in III, inverted T wave in III, and right bundle branch block.

A 54-year-old woman was admitted with a traumatic left tibial fracture to a local hospital. After two days of admission, she developed a sudden onset of dyspnea and chest pain, prompting her to transfer to our tertiary care center for further management. On admission, her vital parameters were as follows: blood pressure 89/51 mmHg, heart rate 104 beats per minute, oxygen saturation 83% on room air, and respiratory rate 28 cycles per minute. Her electrocardiogram showed an SI QIII TIII pattern and right bundle branch block (Figure 1). Laboratory tests showed a white cell count of 10,780/µL, hemoglobin of 11.9 g/dL, platelet count of 293,000/µL, troponin I of 2.82 ng/mL (reference range: <0.120 ng/mL), and serum creatinine of 95 µmol/L. Bedside echocardiography demonstrated massively dilated right atrium and right ventricle, compressed D-shaped left ventricle, and moderate to severe pulmonary hypertension.

**Figure 2:**
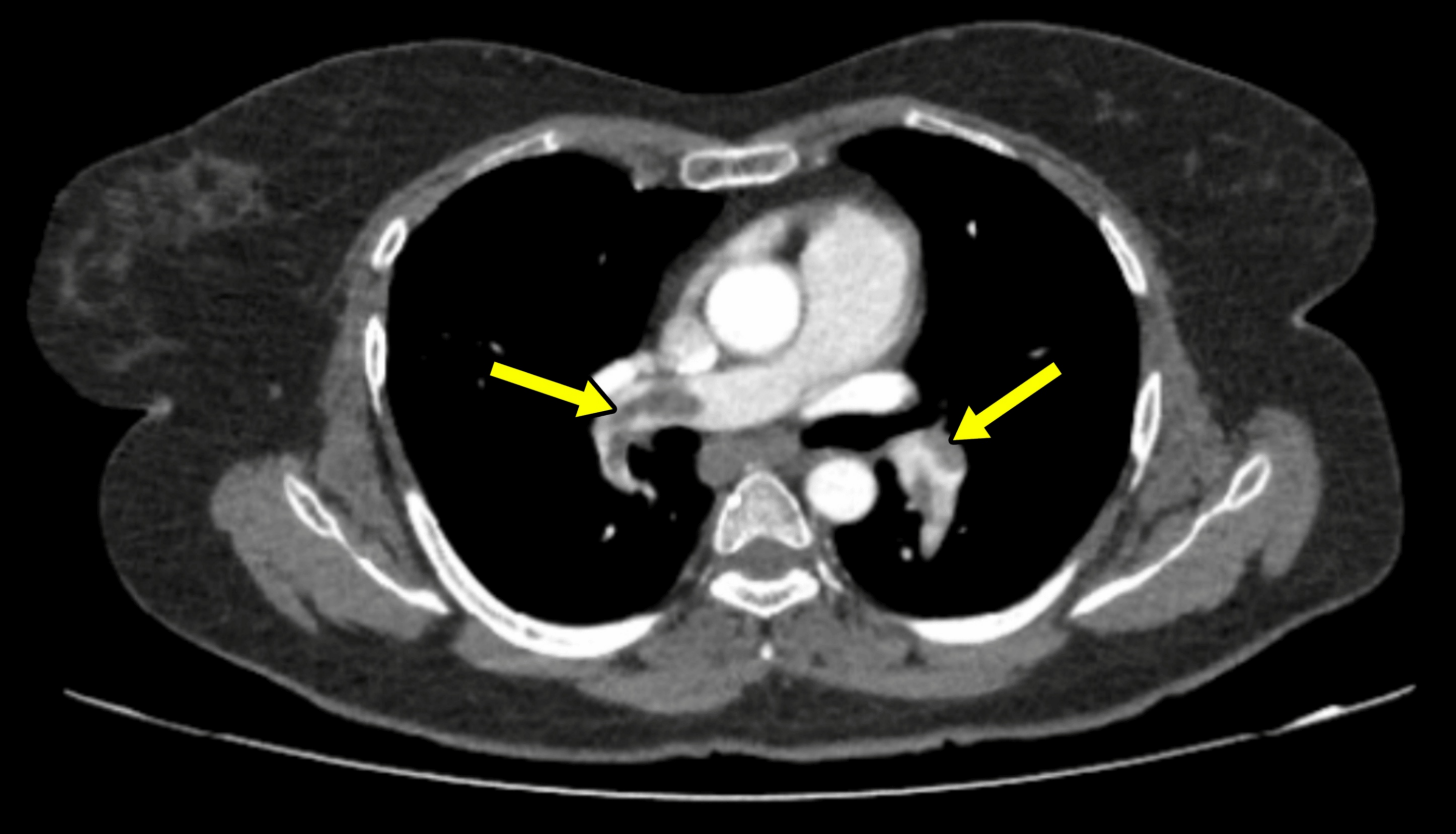
Computed tomography pulmonary angiogram identified filling defects (arrows) in the right and left pulmonary vasculature.

A computed tomography (CT) pulmonary angiogram identified filling defects in both the right and left pulmonary vasculature (Figure 2), confirming the diagnosis of PE. The patient's Simplified Pulmonary Embolism Severity Index (sPESI) score of 3 categorized her as high risk for mortality.

**Figure 3:**
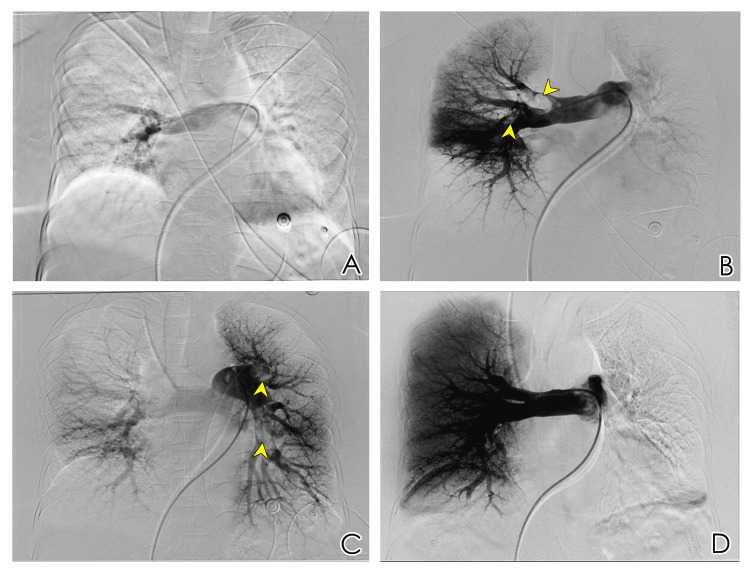
Digital subtraction angiography. (A) Injection of contrast within the right main pulmonary artery showed poor perfusion of the right lung; (B) injection of contrast beyond the thrombus within the right pulmonary artery showed filling defects (arrowheads); (C) injection of contrast within the left main pulmonary artery showed filling defects (arrowheads) and reduced perfusion of the left lung; (D) digital subtraction angiography after 48 hours of the procedure, injection of contrast within the right main pulmonary artery showed improved perfusion of the right lung.

Given the patient's hemodynamic instability, elevated sPESI score, and recent trauma, we opted for catheter-based therapy performed by the interventional radiology team as the initial treatment approach instead of systemic thrombolysis. The procedure was performed under sedation using intermittent pulsed fluoroscopic guidance, and strict aseptic conditions were maintained. Venous access was obtained through the right common femoral vein using a 5F vascular sheath. The inferior vena cava was catheterized, and access was gained to the main pulmonary trunk. Bilateral pulmonary angiography revealed multiple filling defects in the right distal main pulmonary artery extending into the right upper lobar artery, as well as the mid and lower segmental arteries. Filling defects were also noted in the left upper proximal lobar arteries and the anterior division of the left lower lobar artery (Figure 3). Compared to the right lung, perfusion of the left lung was relatively preserved. A guidewire was passed through the thrombus in the right main pulmonary artery to degrade it and push it to the peripheral pulmonary arteries. Suction thrombectomy was attempted but was unsuccessful due to difficulty in effectively aspirating the thrombus.

Following a discussion among the consultant interventional radiologist, consultant physician, and consultant intensivist, the decision was made to proceed with CDT of the right pulmonary artery thrombus. The right pulmonary artery was selected because it demonstrated poorer perfusion than the left, prioritizing the salvage of the most affected side first. The tip of the angled pigtail catheter was positioned in the distal right main pulmonary artery. The catheter was left in place for planned infusion in an intensive care unit (ICU) setting, administering alteplase at a rate of 1 mg/hour for 12 hours. After completion of the alteplase infusion, an unfractionated heparin (UFH) infusion was initiated and titrated based on activated partial thromboplastin time (APTT). The following day, despite clinical improvement, the patient still required oxygen support. The multidisciplinary team decided to administer a second dose of alteplase over the next 12 hours, followed by an infusion of UFH.

A follow-up digital subtraction angiography (DSA) performed 48 hours later showed complete resolution of the thrombus in the right main pulmonary artery and lobar pulmonary arteries (Figure 3D), with significantly improved lung perfusion. The catheter and vascular sheath were subsequently removed. The patient was hemodynamically stable and asymptomatic after the procedure, with no procedure-related complications. As the patient became hemodynamically stable and showed clinical improvement after the right-sided CDT, it was decided not to proceed with further catheter-based therapy on the left pulmonary artery. The patient was started on warfarin, bridged with low-molecular-weight heparin. After five days in our center, she was transferred back to the local hospital for continued care.

## Discussion

Treatment options for PE are tailored according to the clinical classification of the condition (massive, submassive/intermediate-risk, or small) based on the guidelines from the American Heart Association (AHA) and the European Society of Cardiology (ESC). Available therapeutic options include systemic thrombolysis, catheter-based therapies, anticoagulation alone, and surgical intervention. Systemic thrombolysis is often used in massive PE due to its quick action and the urgent need to prevent rapid clinical deterioration [2,4].

This case demonstrates the first experience and successful attempt of CDT in a patient with massive PE and a history of recent trauma at Teaching Hospital Jaffna. It also emphasized the successful adaptation of advanced techniques in our setting. Massive PE is associated with significant morbidity and mortality. The management of massive PE warrants rapid and effective revascularization while reducing bleeding complications. Although systemic thrombolysis has shown the effectiveness of massive PE, it is associated with the risk of bleeding in patients with trauma or surgery due to systemic exposure [3].

In recent years, catheter-based therapies have emerged as an alternative intervention to systemic thrombolysis for the management of massive PE. CDT involves the local administration of thrombolytic agents directly into the pulmonary arteries, thereby reducing systemic exposure and the bleeding risk. Several studies have shown that CDT is an effective and safe option for patients with massive PE. The ULTIMA and SEATTLE II trials showed significant improvements in right ventricular function and pulmonary artery pressure with a lower incidence of major bleeding than systemic thrombolysis [5,6].

A systematic review and meta-analysis by Kuo et al. compared systemic thrombolysis and modern CDT techniques in managing massive PE. The results showed that CDT had similar efficacy in revascularization and hemodynamic stabilization as systemic thrombolysis. Additionally, CDT was associated with fewer major hemorrhagic complications [7]. Engelberger et al. retrospectively investigated the efficacy and safety of fixed low-dose ultrasound-assisted CDT on 52 patients with massive PE. This study found that CDT was associated with rapid improvement in hemodynamic parameters, and major non-fatal bleeding occurred only in two cases (3.8%) [8].

Our patient presented with hemodynamic instability, evidenced by hypotension, hypoxia, and tachycardia. Her echocardiographic findings revealed signs of right ventricular dysfunction and pulmonary hypertension. As she had a recent traumatic fracture, systemic thrombolysis might cause major bleeding events. Suction thrombectomy was initially performed but was unsuccessful. However, CDT successfully revascularized and improved the thrombotic burden. The decision to proceed with CDT was made by a multidisciplinary team, including an interventional radiologist, a physician, and an intensivist specialist. This case emphasizes the successful team-based approach in the management of massive PE. The successful outcome in our patient favored CDT as a safe and effective treatment for massive PE.

This experience marks a significant milestone for Teaching Hospital Jaffna. Future efforts will focus on training and equipping our team to handle similar cases, enhancing our ability to manage high-risk PE patients effectively.

## Conclusions

The CDT represents an effective treatment option for massive PE, particularly those with contraindications to systemic thrombolysis. Our first experience at Teaching Hospital Jaffna demonstrates the feasibility and efficacy of this approach, and it emphasizes the importance of individualized treatment strategies and a multidisciplinary approach in managing massive PE.
